# Biomarkers as predictors of sudden cardiac death in coronary artery disease patients with preserved left ventricular function (*ARTEMIS* study)

**DOI:** 10.1371/journal.pone.0203363

**Published:** 2018-09-18

**Authors:** E. Samuli Lepojärvi, Heikki V. Huikuri, Olli-Pekka Piira, Antti M. Kiviniemi, Johanna A. Miettinen, Tuomas Kenttä, Olavi Ukkola, Juha S. Perkiömäki, Mikko P. Tulppo, M. Juhani Junttila

**Affiliations:** Medical Research Center Oulu, Research Unit of Internal Medicine and Clinical Chemistry, Oulu University Hospital and University of Oulu, Oulu, Finland; Cincinnati Children's Hospital Medical Center, UNITED STATES

## Abstract

**Aims:**

Biomarkers have shown promising results in risk assessment of cardiovascular events. Their role in predicting the risk of sudden cardiac death (*SCD*) is not well established. We tested the performance of several biomarkers in risk assessment for *SCD* in patients with coronary artery disease (*CAD*) and preserved left ventricular function.

**Methods and results:**

The study population consisted of 1,946 *CAD* patients (68% male; mean age 66.9±8.6 yrs; type 2 diabetes (*T2D*) 43%) enrolled in the *ARTEMIS* study. The study subjects underwent examinations with echocardiography and measurement of several biomarkers. The primary endpoint of the study was *SCD*. During the mean follow up of 76±20 months 50 patients experienced SCD. Elevated high sensitive *CRP* (*hs-CRP*, p = 0.001), soluble *ST2* (*sST2*, p<0.001), B-type natriuretic peptide (*BNP*, p<0.001), and highly sensitive TroponinT (*hs-TnT*, p<0.001) predicted the occurrence of *SCD* in univariate analysis. Using the optimal cutoff points, elevated *sST2* (≥27.45ng/mL; hazard ratio [*HR*] 2.7; 95%*CI* 1.4–5.1, p = 0.003) and *hs-TnT* (≥15 ng/mL; *HR* 2.9; 95% *CI* 1.5–5.6, p = 0.002) were the strongest predictors of *SCD* followed by *hs-CRP* (*HR* 2.4; 95% CI 1.3–4.4, p = 0.004) and *BNP* (*HR* 1.9; 95% *CI* 1.0–3.7, p = 0.046) in adjusted analysis. Combination of elevated *hs-TnT* and *sST2* resulted in adjusted *HR* of 6.4 (95% *CI* 2.6–15.5, p<0.001).

**Conclusion:**

Elevated *sST2* and *hs-TnT* predict the occurrence of *SCD* among patients with *CAD* and preserved left ventricular function. The association between *sST2*, *hs-TnT* and *SCD* may be explained by an ongoing myocardial apoptosis followed by fibrosis leading to vulnerability to malignant arrhythmias.

## Introduction

Coronary artery disease (*CAD*) is still the most common cause of death in Western society. Even though the prevention and treatment of *CAD* has improved during the last three decades, the impact of sudden cardiac death (*SCD*) remains prominent[1–3]. The majority of *SCDs* are due to *CAD*, but to date only few clinical or biological markers have succeeded in identifying individuals at risk in an otherwise intermediate risk population of stable *CAD* patients [4]. The risk assessment of *SCD* in *CAD* populations has recently concentrated on markers of heart failure and impaired autonomic nervous system. In addition to left ventricular ejection fraction (*LVEF*), numerous non-invasive *ECG*-derived methodologies have been proposed to risk stratify patients for *SCD*. Among them, Holter analysis including heart rate variability and turbulence are commonly recommended for non-invasive risk stratification[5]. Although these markers have been shown to predict *SCD*, the majority of *SCD* victims are not identified by these measures. Feasible markers for *SCD* risk assessment in intermediate risk populations such as *CAD* patients with preserved left ventricular function are lacking. B-type natriuretic peptide (*BNP*) and high-sensitive C-reactive protein (*hs-CRP*) have provided some prognostic information in various populations, and recently, highly sensitive troponin (*hs-TnT*) and markers of cardiac fibrosis, such as *sST2* and galectin-3, have shown promising results in risk assessment of cardiovascular events in patients with *CAD* and congestive heart failure (*CHF*)[6–12]. The occurrence of ventricular arrhythmias is dependent on re-entry formation, and the key factors in this phenomenon are areas of slow conduction. The current hypothesis assumes that these areas are a result of accumulation of fibrosis caused by a myocardial infarction or, more commonly, by repeated episodes of transient ischemia. In many cases, this accumulation is heterogeneous in nature and does not form a stable local scar which does not conduct electricity, but an area where conduction is slow. This fibrosis accumulation is ultimately assumed to lead to reduction of systolic function, but the majority of patients develop concomitant muscle hypertrophy which delays the occurrence of pump failure. On the other hand, there is a risk for arrhythmias. The aim of the study was to test the performance of several biomarkers in risk assessment for *SCD* in *CAD* patients with preserved *LV* function. Ultimately, the goal was to find biomarkers which would coincide with the pathological pathway of fibrosis accumulation and arrhythmia risk elevation.

## Methods

The study population consisted of 1,946 patients with stable *CAD* from the *ARTEMIS* (Innovation to Reduce Cardiovascular Complications of Diabetes at the Intersection) study conducted in the Division of Cardiology of Oulu University Hospital (Oulu, Finland), ClinicalTrials.gov (Identifier NCT01426685). The goal of *ARTEMIS* study is to study the prognostic value of several traditional and novel cardiovascular risk markers in patients with stable *CAD* during a 5-year follow-up period. The design has been previously described in detail[9,13]. The patients were recruited to a thorough risk assessment from a consecutive series of patients referred to coronary angiography in the Division of Cardiology of Oulu University Hospital (Oulu, Finland) 3 to 6 months after the angiography. Most patients had either percutaneous coronary angioplasty or coronary artery bypass during or right after the index procedure. Almost half of the patients had experienced an acute coronary event >3 months before the enrollment into the risk profile study. Exclusion criteria were: age <18 years or >85 years, New York Heart Association or Canadian Cardiovascular Society class IV, significant valvular disease, permanent pacemaker or permanent or planned implantable cardioverter defibrillator implantation (*LV* ejection fraction <35%), end-stage renal failure needing dialysis, life expectancy <1 year. Patients who were psychologically or physically (due to any other illness) unfit for participation in the study were also excluded from this study. The study was performed according to the Declaration of Helsinki, and the local committee of research ethics of the Northern Ostrobothnia Hospital District approved the protocol, and all the subjects gave written informed consent.

Laboratory samples were obtained after 12-hour overnight fast using standardized methods. Subjects without known diabetes had an oral glucose tolerance test. *T2D*, impaired glucose tolerance, and impaired fasting glycemia were verified according to current criteria of the World Health Organization[14]. Patients who were on antidiabetic medication based on previous diagnosis of diabetes were also classified as having *T2D*. Blood samples and urine samples were obtained for the analysis of renal function (albumin-to-creatinine ratio), inflammation markers (*hs-CRP*), lipids, glycated hemoglobin (*HbA1c*), and cardiac markers (*BNP* and *hs-TnT*), and they were analyzed by the hospital laboratory. The concentrations of *BNP* and *hs-TnT* were determined from plasma samples (ADVIA Centaur XP, Siemens Healthcare Diagnostics and MODULAR ANALYTICS, Roche Diagnostics, respectively). The concentrations of *hs-CRP*, galectin-3, and *sST2* were determined from serum samples (BN Prospec System, Siemens Healthcare Diagnostics; Human ST2/IL-1 R4 Quantikine ELISA, R&D Systems Inc., Minneapolis, Minnesota; BG Medicine, Waltham, Massachusetts, respectively). Echocardiography was performed according to the American Society of Echocardiography guidelines. Left ventricular (*LV*) mass was calculated using the formula recommended by the American Society of Echocardiography (LV mass = 0.8 × [1.04 {(LVIDd + PWTd + SWTd)^3^ –(LVIDd)^3^}] + 0.6 g). *LV* mass index was calculated by dividing LV mass by body surface area. Patients with LVEF <35% who had an indication for prophylactic implantable cardioverter-defibrillator were excluded from the consecutive series. *SYNTAX* Score was calculated by three experienced interventional cardiologists using the Web-based calculator version 2.11 on the *SYNTAX* Score Web site (www.syntaxscore.com).

The primary endpoint of the *ARTEMIS* study is *SCD*, including aborted cardiac arrests. The occurrence of *SCD* and aborted cardiac arrests was defined by an endpoint committee (*HVH* and *MJJ*) from death certificates including autopsy reports, hospital records, and contacts with next of kin. Only probable or possible sudden deaths were classified as *SCDs*, including witnessed sudden death within one hour after the onset of symptoms, or within 24 hours of when the victim was last seen alive without any evidence of non-cardiac cause of death at autopsy. The secondary endpoint of the study was defined as all-cause mortality (*ACM*).

### Statistical analyses

Values are expressed as mean (+/-*SD*), median (interquartile range) or the number of subjects (%). Two-tailed t-test for independent samples, Mann-Whitney U-test (if non-Gaussian distribution of the data) or chi-square were used to compare the survivors with the groups of *SCD* and *ACM*. Receiver operating characteristic (*ROC*) was used to establish the optimal cutoff value for the novel biomarkers using *SCD* as endpoint. The cutoff was set to the value that provided the maximum sum of sensitivity and specificity, sensitivity being at least 20% but less than 50% of patients having positive result. The prognostic significances of biomarkers were assessed according to the recommendations by Hlatky et al. [15]. These recommendations suggest that when a new risk marker is introduced in a system of multiple risk factors the ability to improve the model (which is generated with standard risk factors) should be assessed. In our study, attempts to assess the additive risk value of biomarkers have been made with C-statistics and integrated discrimination improvement (*IDI*) calculation. Hazard ratios (*HR*) with 95% confidence intervals (*CI*) were calculated by univariate Cox regression analysis for each risk marker. Thereafter, the predictive value of each biomarker was assessed in multivariate Cox regression where age, sex, body mass index, Canadian Cardiovascular Society grading of angina pectoris, left ventricular ejection fraction, albumin-creatinine ratio, estimated glomerulus filtration rate (*eGFR*), *HbA1c*, and *T2D* were entered in the model as continuous variables when applicable. The covariates were selected based on clinical significance and relevant differences found in characteristics between the groups with and without *SCD*. Multimarker Cox regression models were also established for the most significant biomarkers followed by further adjustments for potential confounders. Competing risks survival analyses were also conducted using approaches of *SCD* vs.non-sudden cardiac death.

Due to the varying follow-up time, discrimination and reclassification analyses were based on individual time-dependent estimated risk for *SCD* that was obtained by multivariate Cox regression analyses. The added discrimination of the risk markers was assessed by the C-index and the integrated discrimination index (*IDI*). Categorical and continuous net reclassification index (*NRI*; risk levels: 0–<6, 6–<20 and ≥20%) were also calculated[16]. Kaplan-Meier analysis with log rank analysis was used to describe the SCD-free survival in low- and high-risk groups. The data were analyzed using SPSS software (IBM SPSS Statistics 21, IBM Corp., New York, USA) and R Statistics (3.3.1, The R Foundation for Statistical Computing, Vienna, Austria). A p-value <0.05 was considered statistically significant.

## Results

The characteristics of the patients are presented in Table 1. Mean follow-up time was 76±20 months during which 205 deaths occurred of which 99 were determined to be of cardiac cause. Among cardiac deaths 50 *SCDs*, including 8 aborted cardiac arrests, were observed. Non-sudden cardiac death occurred in 44 subjects, which include pump failure and heart failure deaths. The concentrations of biomarkers, except for galectin-3, were higher in the patients who suffered *SCD* than those without the primary endpoint (Table 1).

**Table 1:** Characteristics of coronary artery disease patient group of survivors, sudden cardiac death (SCD) and all-cause mortality (ACM).

	Survivors	SCD	ACM
	n = 1740	n = 50	n = 205
Age (years)	66 (9)	69 (7)*	72 (8)‡
Sex (male)	1166 (67%)	39 (78%)	161 (79%)†
BMI (kg/m^2^)	28.3 (4.5)	28.7 (4.8)	28.5 (5.1)
Systolic BP (mmHg)	147 (24)	146 (22)	146 (27)
Diastolic BP (mmHg)	81 (11)	80 (11)	79 (13)
Smokers	146 (8%)	7 (14%)	21 (10%)
History of AMI (n)	826 (48%)	28 (56%)	103 (50%)
History of PCI/CABG (n)	1386 (80%)	42 (84%)	158 (77%)
Syntax score	0 (0–5)	3 (0–8)	2 (0.9)‡
CCS class (n)		‡	‡
1	1000 (59%)	15 (31%)	79 (39%)
2	594 (35%)	26 (54%)	80 (39%)
3	113 (7%)	7 (15%)	41 (20%)
Type of Glucose Metabolism Disorder		†	‡
Impaired Glucose Tolerance (n)	280 (16%)	7 (14%)	34 (17%)
Impaired Fasting Glucose (n)	98 (6%)	2 (4%)	5 (2%)
Diabetes (n)	709 (41%)	34 (68%)	124 (61%)
Duration of diabetes (months)	60 (24–132)	54 (21–213)	96 (24–228)†
**Medication** (n)			
β-blocker	1518 (87%)	45 (90%)	186 (91%)
ACEi/ARB	1182 (68%)	37 (74%)	148 (72%)
CCB	422 (24%)	11 (22%)	52 (25%)
Diuretics	554 (32%)	26 (52%)†	112 (55%)‡
Lipids	1598 (92%)	44 (88%)	179 (87%)*
**Echogardiography**			
LVEF (%)	64 (9)	58 (14)‡	61 (13)‡
LVMI (g/m^2^)	107 (27)	118 (30)†	118 (31)‡
**Laboratory analyses**			
HbA_1c_ (%)	6.3 (0.9)	6.9 (1.7)‡	6.8 (1.5)‡
Total cholesterol (mmol/L)	4.0 (0.9)	4.4 (1.1)†	4.0 (1.0)
HDL cholesterol (mmol/L)	1.3 (0.3)	1.2 (0.4)	1.2 (0.3)*
LDL cholesterol (mmol/L)	2.3 (0.8)	2.6 (1.0)†	2.3 (0.9)
Triglycerides (mmol/L)	1.2 (0.9–1.7)	1.4 (0.9–1.9)	1.2 (0.9–1.7)
eGFR (mL/min)	95 (34)	87 (33)	81 (35)‡
U-Alb/Crea	0.8 (0.6–1.3)	1.1 (0.9–2.4)‡	1.3 (0.7–2.6)‡
hs-CRP (mg/L)	0.9 (0.5–1.9)	1.8 (0.7–6.0)†	1.4 (0.7–3.7)‡
hs-TnT (ng/L)	8 (5–12)	15 (7–24)‡	15 (9–23)‡
BNP (ng/L)	47 (24–87)	89 (36–158)‡	91 (36–199)‡
sST2 (ng/mL)	16.3 (12.8–21.5)	20.3 (15.2–30.5)‡	21.5 (15.4–29.3)‡
Galectin-3 (ng/mL)	11.0 (9.1–13.4)	12.0 (9.0–15.6)	13.0 (10.4–18.6)‡

Values are means (SD), number of cases (%) or median (interquartile range).

Abbreviations: BMI = body mass index, BP = blood pressure, AMI = acute myocardial infarction, CCS = Canadian Cardiovascular Society grading of angina pectoris, ACEi = angiotensin converting enzyme, ARB = angiotensin II receptor blocker, CCB = calcium channel blocker, LVEF = left ventricle ejection fraction, LVMI = left ventricle mass index, HbA_1c_ = Glycosylated Hemoglobin, Type A1C, eGFR = estimated glomerulus filtration rate, hs-CRP = high-sensitive C-reactive protein, hs-TnT = highly sensitive Troponin T, BNP = B-type natriuretic peptide, sST2 = soluble ST2.

* p<0.05

† p<0.01 and

‡ p<0.001

Elevated *sST2* and *hs-TnT* were the most powerful predictors of the primary endpoint in the univariate analyses and also in multivariate analyses with 2.7- and 2.9-fold risk for *SCD* (p = 0.003 and p = 0.002), respectively (Table 2). They also were the most significant risk markers in the crude and adjusted multimarker Cox regression, being significantly associated with SCD independently of *hs-CRP* and *BNP* (Table 3). When the two most powerful biomarkers, i.e. *sST2* and *hs-TnT*, were combined, elevated concentrations in both biomarkers yielded a hazard ratio (*HR*) of 6.4 (2.6–15.5, p<0.001) compared to those with both at normal level in multivariate Cox analysis (Table 2). *Hs-TnT*, *hs-CRP*, *BNP* and sST2 were also significant predictors of all-cause mortality in multivariate analysis (p<0.001 for all, Table 2).

**Table 2:** Univariate and multivariate Cox regression analysis of novel biomarkers as predictor of sudden cardiac death and all-cause mortality in all patients.

		Sudden cardiac death			All-cause mortality		
			Univariate	Multivariate		Univariate	Multivariate
	n (yes/no)	Events (yes/no)	Hazard Ratio (95% CI)	Hazard Ratio (95% CI)	Events (yes/no)	Hazard Ratio (95% CI)	Hazard Ratio (95% CI)
hs-TnT ≥ 15 ng/L	404/1541	27/23	5.0 (2.8–8.7)‡	2.9 (1.5–5.6)†	109/96	4.9 (3.7–6.4)‡	2.4 (1.7–3.3)‡
hs-CRP ≥ 1.73 mg/L	585/1353	24/26	2.6 (1.5–4.5)†	2.4 (1.3–4.4)†	95/110	2.1 (1.6–2.7)‡	2.3 (1.7–3.1)‡
BNP ≥ 88 ng/L	536/1410	24/26	3.2 (1.8–5.5)‡	1.9 (1.0–3.7)*	107/98	3.2 (2.4–4.2)‡	1.9 (1.4–2.6)‡
sST2 ≥ 27.4 ng/mL	227/1718	32/18	4.5 (2.5–8.1)‡	2.7 (1.4–5.1)‡	63/142	3.6 (2.7–4.9)‡	2.6 (1.9–3.6)‡
hs-TnT < 15 ng/L and sST2 < 27.4 ng/mL	1399/546	17/33	Reference	Reference	76/129	Reference	Reference
hs-TnT ≥ 15 ng/L and sST2 < 27.4 ng/mL	319/1626	15/35	4.2 (2.1–8.5)‡	2.6 (1.2–5.7)*	66/139	4.2 (3.0–5.8)‡	2.1 (1.4–3.0)‡
hs-TnT < 15 ng/L and sST2 ≥ 27.4 ng/mL	142/1803	6/44	3.6 (1.4–9.0)†	2.4 (0.9–6.8)	20/185	2.7 (1.6–4.4)‡	2.1 (1.3–3.5)†
hs-TnT ≥ 15 ng/L and sST2 ≥ 27.4 ng/mL	85/1860	12/38	13.8 (6.6–29.0)‡	6.4 (2.6–15.5)‡	43/182	11.6 (8.0–16.8)‡	5.6 (3.6–8.6)‡

**Abbreviations:** hs-TnT = highly sensitive troponin T, sST2 = soluble ST 2

Adjusted for age, sex, body mass index, Canadian Cardiovascular Society grading of angina pectoris, left ventricular ejection fraction, estimated glomerulus filtration rate, albumin-creatinine-ratio, glycated hemoglobin, and diabetes. Seventy patients excluded due to missing covariates.

* p<0.05

† p<0.01

‡ p<0.001

**Table 3:** Multimarker Cox regression model for the most significant biomarkers predicting sudden cardiac death and all-cause mortality.

	Sudden cardiac death		All-cause mortality	
	Multimarker model	Adjusted multimarker model	Multimarker model	Adjusted multimarker model
	Hazard Ratio (95% CI)	Hazard Ratio (95% CI)	Hazard Ratio (95% CI)	Hazard Ratio (95% CI)
hs-TnT ≥ 15 ng/L	3.2 (1.8–5.8)‡	2.4 (1.2–4.7)*	3.3 (2.5–4.4)‡	2.0 (1.4–2.7)‡
hs-CRP ≥ 1.73 mg/L	1.9 (1.0–3.3)*	2.0 (1.1–3.6)*	1.6 (1.2–2.1)†	1.8 (1.3–2.4)‡
BNP ≥ 88 ng/L	2.1 (1.2–3.8)*	1.7 (0.9–3.3)	2.2 (1.7–3.0)‡	1.7 (1.2–2.3)†
sST2 ≥ 27.4 ng/mL	2.8 (1.5–5.2)†	2.1 (1.1–4.1)*	2.4 (1.7–3.3)‡	2.1 (1.5–3.0)‡

**Abbreviations:** hs-TnT = highly sensitive troponin T, hs-CRP = highly sensitive C-reactive protein, BNP = B-type natriuretic peptide, sST2 = soluble ST 2

Adjusted for age, sex, body mass index, Canadian Cardiovascular Society grading of angina pectoris, left ventricular ejection fraction, estimated glomerulus filtration rate, albumin-creatinine-ratio, glycated hemoglobin, and diabetes. 70 patients excluded due to missing covariates.

* p<0.05

† p<0.01

‡ p<0.001

Elevated *hs-TnT* and *sST2* were associated with *SCD* regardless of competing risk for non-sudden cardiac death in univariate analysis (*HR*: 4.8, 95%*CI*: 2.7–8.4 for *hs-TnT* and *HR*: 3.9, 95%*CI*: 2.1–7.2 for *sST2*, p<0.001 for both) and after adjustment for the established risk markers (*HR*: 2.8, 95%*CI*: 1.4–5.5, p = 0.003 for *hs-TnT* and *HR*: 2.5, 95%*CI*: 1.2–5.0, p = 0.012 for *sST2*). Univariate and adjusted *HR*s for the non-sudden cardiac death from the competing risks analysis were 7.5 (95%*CI*: 4.2–13.6, p<0.001) and 2.3 (95%*CI*: 1.1–4.9, p = 0.027) for *hs-TnT*, and 4.0 (95%*CI*: 2.2–7.3, p<0.001) and 2.7 (95%*CI*: 1.4–5.1, p = 0.003) for *sST2*, respectively.

**Fig 1:**
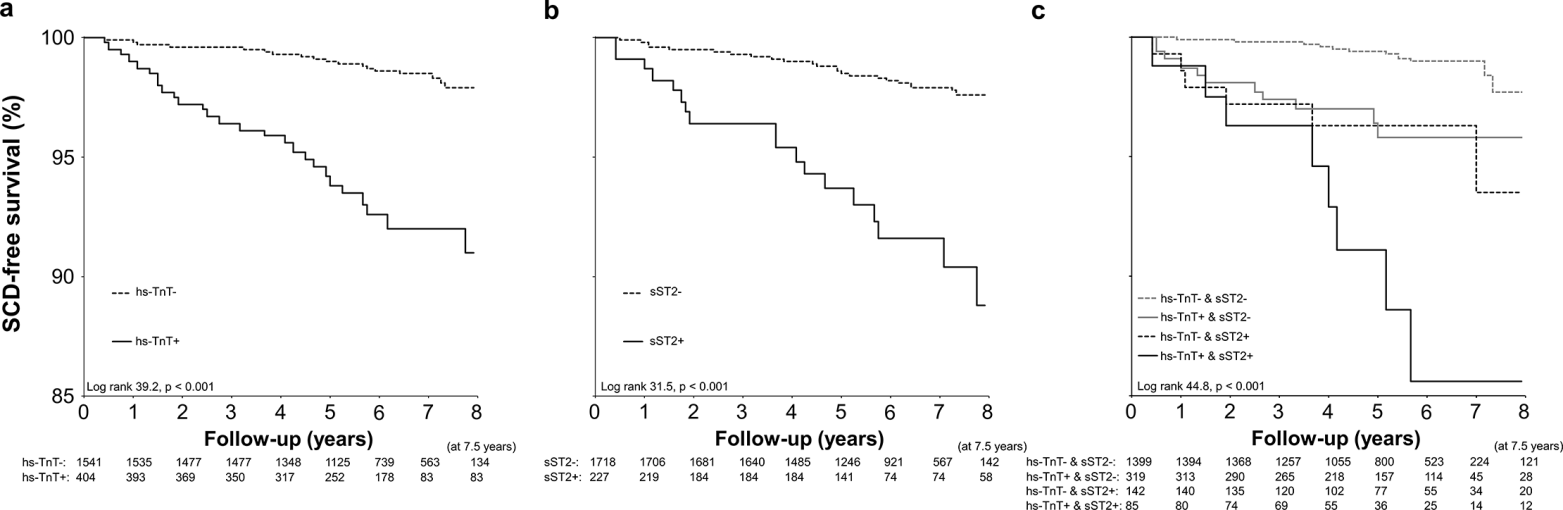
Kaplan-Meier curves of sudden cardiac death (SCD) free survival of patients with elevated levels of high sensitive troponin (hs-TnT) (panel A), soluble ST2 (sST2) (panel B), or both (panel C). + indicates elevated values and—indicates value that is not elevated when optimal cutoffs are used.

The addition of *hs-TnT* to the established model for *SCD* improved discrimination (*IDI*: 0.012; 95% *CI*: 0.001–0.023, p = 0.035) as well as the classification by continuous (0.488; 95%*CI*: 0.204–0.772. p<0.001) but not categorical *NRI* (0.064; 95%*CI*: -0.078–0.206, p = 0.38). The inclusion of *sST2* in the risk model did not improve discrimination (*IDI*: 0.011, 95%*CI*: -0.006–0.029. p = 0.22) and improved the classification of the patients only when analyzed by continuous *NRI* (0.349, 95%*CI*: 0.080–0.618, p = 0.011). When both elevated *hs-TnT* and *sST2* were added to the established model, C-index improved from 0.732 (95%CI: 0.665–0.799) to 0.780 (95%CI: 0.720–0.839); the *IDI* being significant (0.023; 95%CI: 0.002–0.043, p = 0.028). Continuous (0.400, 95%CI: 0.114–0.686, p = 0.006), but not categorical *NRI* (0.133; 95%CI: -0.033–0.300, p = 0.12), indicated improved classification of the cases. The Kaplan-Meier curves of elevated *hs-TnT*, *sST2*, and their combination are presented in the Fig 1.

In *CAD* patients with *T2D*, abnormal *hs-TnT* and *hs-CRP* were the only variables that predicted *SCD* (34 events, 4.1%) in the multivariate analysis (hazard ratio: 3.0; 95%CI: 1.2–7.2, p = 0.014 and hazard ratio 2.6; 95% *CI*: 1.2–5.4, p = 0.012), whereas among those without *T2D*, only abnormal *sST2* predicted the primary outcome (16 events, 1.4%; hazard ratio: 7.2; 95%*CI*: 2.4–22.0, p = 0.001). *Hs-TnT*, *hs-CRP*, *BNP* and *sST2* were significant predictors of all-cause mortality in *CAD* patients with (2.8-, 2.8-, 1.5 and 2.6-fold adjusted risk, respectively, p<0.05 for all) and without *T2D* (1.9-, 1.6-, 2.6 and 3.0-fold adjusted risk, respectively, p<0.05 for all).

## Discussion

This study showed that measurement of biomarkers from blood samples provides information on *SCD* risk among the patients with *CAD* and preserved left ventricular function. Soluble *ST2* and *hs-TnT* were the most powerful predictors after adjustments with several clinical and laboratory variables as well as *LVEF*. A combination of elevated concentrations of both sST2 and hs-TnT yielded high predictive accuracy in *SCD* prediction in this population.

The present study aimed to focus on the risk assessment of *SCD* among *CAD* patients with preserved left ventricular function, since the majority of *SCD* victims have a heart disease with normal or mildly impaired cardiac function[17,18]. Furthermore, the majority of prior risk stratification studies have focused on patients with *CHF* or on those with a recent acute myocardial infarction. This has raised the question of whether any of the risk stratification methods can be improved in order to develop appropriate preventive strategies for this large number of potential victims of *SCD* included in the present study.

### Biomarkers in risk prediction

All biomarkers included in this study have provided prognostic information in various patient populations in prior studies, but the utility of these variables in prediction of *SCD* risk has not been thoroughly studied. To our knowledge, *BNP* is the only biomarker that has been shown to predict SCD after acute myocardial infarction[19]. The other biomarkers, such as *sST2*, *hs-TnT*, galectin-3 and *hs-CRP*, have predicted all-cause and cardiac mortality among patients with heart failure, stable *CAD* and even in general population[6,8,10,20,21], but only one of these studies focused specifically on the occurrence of *SCD* in patients with *CHF* [11] while none focused on the occurrence of *SCD* in patients with preserved left ventricular function. Multimarker strategy, i.e. combination of two or more risk variables, has been shown to improve the prediction of cardiovascular events in previous studies[10,22]. Combination of elevated *sST2* and *hs-TnT* in the present population also provided the most powerful information on the risk of *SCD*.

### Potential mechanisms of association between sST2, hs-TnT and SCD

Soluble *ST2* and *hs-TnT* were the strongest predictors of *SCD* in the present study. Soluble *ST2* is a member of the interleukin-1 receptor family and circulating *sST2* concentrations are believed to reflect cardiovascular stress and cardiac fibrosis[23,24]. Soluble *ST2* is elevated in *CHF*, older age and in diabetes[25]. It has also been shown that genetic factors explain up to 45% of the *sST2* concentrations in general population[26]. *Hs-TnT* is a novel marker reflecting ongoing myocyte death and apoptosis without any evidence of an acute ischemic event. Elevated levels of *hs-TnT* have been associated with higher risk of incident coronary event, hospitalization due to heart failure, cardiac mortality, cardiovascular mortality, stroke and cancer[6–8,27,28]. In our prior pilot study of the *ARTEMIS* population, we found that *hs-TnT* is a strong predictor of a composite endpoint of cardiac death and hospitalization for heart failure during a 2-year follow-up[9].

The mechanism why both *sST2* and *hs-TnT* predicted *SCD* remains speculative. These two biomarkers obviously reflect partly different aspects of cardiovascular stress and tissue damage leading to untoward cardiac events, either progressive heart failure or occurrence of *SCD* even without prior evidence of left ventricular systolic dysfunction. It can be hypothesized that elevated *hs-TnT* is a marker of ongoing myocyte loss and elevated *sST2* reflects the consequent cardiac replacement fibrosis as a result of cell death, which eventually creates a substrate for fatal arrhythmia. Fibrotic scarring has been shown to correlate strongly with an increased incidence of arrhythmias and *SCD*[29]. Our results thus suggest that *CAD* patients who would otherwise be at low to intermediate risk of *SCD* can be included in a higher risk group with biomarkers of inducted replacement fibrosis accumulation. This paradigm explains partly why some patients with stable *CAD* and normal *LV* function enter a downslope in prognosis through continuous cell death and formation of replacement fibrosis. This scenario would eventually end up in decreased *LV* systolic function, but we do not currently often encounter *CAD* patients with decreasing LV function. On the other hand, the number of patients with some form of heart failure with preserved *EF* is increasing. Thus more patients with accumulated fibrosis are at risk of lethal arrhythmias than lethal pump failure.

### Potential limitations

The *ARTEMIS* study design including a relatively large number of patients with *T2D* is a potential limitation of the present study. The power of the study does not allow risk prediction separately for non-diabetic patients. Therefore, it remains uncertain whether the present results are applicable only to *CAD* patients with *T2D* but not to non-diabetic patients. Further studies should focus also on non-diabetic patients with preserved left ventricular function. A larger sample size is needed for this purpose, since the incidence of *SCD* is much smaller in non-diabetic patients with *CAD* as compared to their diabetic counterparts[30]. It is clear that these results are not strong enough for interventional therapy with *ICD*, but the hypothesis of ongoing fibrosis accumulation in *CAD* patients would hopefully result in more precise examination methods and more sophisticated intervention methods in the future. Additionally, in our analysis we used low sensitivity *ELISA* assays which are not approved for clinical diagnostics and can be viewed as a potential limitation. Also the number of subjects in this study prevented us from dividing the population to training and test cohorts. Unfortunately we do not have sufficient interim information on *EF%* development in this study and thus cannot show whether the biomarker levels are specifically associated with *SCD* or deterioration of *LV* systolic function.

### Conclusions

The present study showed that analysis of serum biomarkers can provide important information on the risk of *SCD* in *CAD* patients with preserved left ventricular systolic function. The multimarker strategy appeared very useful in this respect. Future research should focus on preventive strategies among patients with elevated levels of biomarkers, especially both *hs-TnT* and *sST2*. Examples include angiotensin-converting enzyme inhibitors, aldosterone antagonists, nesiritide, emerging antifibrotic therapies[31], or perhaps novel pharmacological antidiabetic strategies[32].

## Supporting information

S1 File ARTEMIS trial data. (XLSX)

## References

[1] Townsend N, Nichols M, Scarborough P, Rayner M. Cardiovascular disease in Europe—Epidemiological update 2015. *Eur Heart J* 2015;36:2696–2705. 10.1093/eurheartj/ehv428 26306399

[2] Nichols M, Townsend N, Scarborough P, Rayner M. Trends in age-specific coronary heart disease mortality in the European Union over three decades: 1980–2009. *Eur Heart J* 2013;34:3017–3027. 10.1093/eurheartj/eht159 23801825 PMC3796269

[3] Go AS, Mozaffarian D, Roger VL, Benjamin EJ, Berry JD, Borden WB et al Executive summary: Heart disease and stroke statistics-2013 update: A Report from the American Heart Association. *Circulation* 2013;127:143–152. 10.1161/CIR.0b013e318282ab8f 23283859

[4] Myerburg RJ, Junttila MJ. Sudden cardiac death caused by coronary heart disease. Circulation. 2012;125:1043–52. 10.1161/CIRCULATIONAHA.111.023846 22371442

[5] Goldberger JJ, Cain ME, Hohnloser SH, Kadish AH, Knight BP, Lauer MS et al American Heart Association/American College of Cardiology Foundation/Heart Rhythm Society Scientific Statement on Noninvasive Risk Stratification Techniques for Identifying Patients at Risk for Sudden Cardiac Death. A Scientific Statement From the American Heart Association Council on Clinical Cardiology Committee on Electrocardiography and Arrhythmias and Council on Epidemiology and Prevention. *J Am Coll Cardiol* 2008;52:1179–1199. 10.1016/j.jacc.2008.05.003 18804749

[6] Omland T, Pfeffer MA, Solomon SD, De Lemos JA, Røsjø H, Benth JŠ et al Prognostic value of cardiac troponin i measured with a highly sensitive assay in patients with stable coronary artery disease. *J Am Coll Cardiol* 2013;61:1240–1249. 10.1016/j.jacc.2012.12.026 23414791

[7] Omland T, De Lemos JA, Sabatine MS, Christophi CA, Rice MM, Jablonski KA et al A sensitive cardiac troponin T assay in stable coronary artery disease. *N Engl J Med* 2009;361:2538–2547. 10.1056/NEJMoa0805299 19940289 PMC2997684

[8] Oluleye OW, Folsom AR, Nambi V, Lutsey PL, Ballantyne CM. Troponin T, B-type natriuretic peptide, C-reactive protein, and cause-specific mortality. *Ann Epidemiol* 2013;23:66–73. 10.1016/j.annepidem.2012.11.004 23228375 PMC3543509

[9] Lepojärvi ES, Piira O, Kiviniemi AM, Miettinen JA, Kenttä T, Ukkola O et al Usefulness of Highly Sensitive Troponin as a Predictor of Short-Term Outcome in Patients With Diabetes Mellitus and Stable Coronary Artery Disease (from the ARTEMIS Study). *Am J Cardiol* 2016;117:515–521. 10.1016/j.amjcard.2015.11.038 26739392

[10] Dieplinger B, Egger M, Haltmayer M, Kleber ME, Scharnagl H, Silbernagel G et al Increased soluble ST2 predicts long-term mortality in patients with stable coronary artery disease: Results from the ludwigshafen risk and cardiovascular health study. *Clin Chem* 2014;60:530–540. 10.1373/clinchem.2013.209858 24401186

[11] Pascual-Figal DA, Ordoñez-Llanos J, Tornel PL, Vázquez R, Puig T, Valdés M, Cinca J et al Soluble ST2 for Predicting Sudden Cardiac Death in Patients With Chronic Heart Failure and Left Ventricular Systolic Dysfunction. *J Am Coll Cardiol* 2009;54:2174–2179. 10.1016/j.jacc.2009.07.041 19942089

[12] De Boer RA, Lok DJA, Jaarsma T, Van Der Meer P, Voors AA, Hillege HL et al Predictive value of plasma galectin-3 levels in heart failure with reduced and preserved ejection fraction. *Ann Med* 2011;43:60–68. 10.3109/07853890.2010.538080 21189092 PMC3028573

[13] Karjalainen JJ, Kiviniemi AM, Hautala AJ, Piira O-P, Lepojärvi ES, Peltola MA et al Determinants and prognostic value of cardiovascular autonomic function in coronary artery disease patients with and without type 2 diabetes. *Diabetes Care* 2014;37:286–294. 10.2337/dc13-1072 23959565

[14] WHO. Definition, diagnosis and classification of diabetes mellitus and its complicatioins, Part 1: Diagnosis and Classification of Diabetes Mellitus Geneva: WHO, Department of Noncommunicable Disease Surveillance 1999;

[15] Hlatky MA, Greenland P, Arnett DK, Ballantyne CM, Criqui MH, Elkind MSV et al Criteria for evaluation of novel markers of cardiovascular risk: A scientific statement from the American heart association. *Circulation* 2009;119:2408–2416. 10.1161/CIRCULATIONAHA.109.192278 19364974 PMC2956982

[16] Pencina MJ, D'Agostino RB, Steyerberg EW. Extensions of net reclassification improvement calculations to measure usefulness of new biomarkers. Stat Med 2011;30:11–21. 10.1002/sim.4085 21204120 PMC3341973

[17] Wellens HJJ, Schwartz PJ, Lindemans FW, Buxton AE, Goldberger JJ, Hohnloser SH et al Risk stratification for sudden cardiac death: Current status and challenges for the future. *Eur Heart J* 2014;35:1642–1651. 10.1093/eurheartj/ehu176 24801071 PMC4076664

[18] Mäkikallio TH, Barthel P, Schneider R, Bauer A, Tapanainen JM, Tulppo MP et al Prediction of sudden cardiac death after acute myocardial infarction: Role of Holter monitoring in the modern treatment era. *Eur Heart J* 2005;26:762–769. 10.1093/eurheartj/ehi188 15778204

[19] Tapanainen JM, Lindgren KS, Mäkikallio TH, Vuolteenaho O, Leppäluoto J, Huikuri HV. Natriuretic peptides as predictors of non-sudden and sudden cardiac death after acute myocardial infarction in the beta-blocking era. *J Am Coll Cardiol* 2004;43:757–763. 10.1016/j.jacc.2003.09.048 14998613

[20] Everett BM, Brooks MM, Vlachos HEA, Chaitman BR, Frye RL, Bhatt DL. Troponin and Cardiac Events in Stable Ischemic Heart Disease and Diabetes. *N Engl J Med* 2015;373:610–620. 10.1056/NEJMoa1415921 26267622 PMC4627639

[21] Lok DJ, Lok SI, Bruggink-André De La Porte PW, Badings E, Lipsic E, Van Wijngaarden J et al Galectin-3 is an independent marker for ventricular remodeling and mortality in patients with chronic heart failure. *Clinical Research in Cardiology* 2013;102:103–110. 10.1007/s00392-012-0500-y 22886030

[22] Wang TJ, Wollert KC, Larson MG, Coglianese E, McCabe EL, Cheng S et al Prognostic Utility of Novel Biomarkers of Cardiovascular Stress: The Framingham Heart Study. *Circulation* 2012;126:1596–1604. 10.1161/CIRCULATIONAHA.112.129437 22907935 PMC3656719

[23] Pascual-Figal DA, Januzzi JL. The Biology of ST2: The International ST2 Consensus Panel. *Am J Cardiol* 2015;115:3B–7B. 10.1016/j.amjcard.2015.01.034 25665766

[24] Lepojärvi ES, Piira O-P, Pääkkö E, Lammentausta E, Risteli J, Miettinen JA et al Serum PINP, PIIINP, galectin-3, and ST2 as surrogates of myocardial fibrosis and echocardiographic left venticular diastolic filling properties. *Front Physiol* 2015;6 10.3389/fphys.2015.00200 PMC4499700 26217237

[25] Mueller T, Jaffe AS. Soluble ST2—Analytical Considerations. *Am J Cardiol* 2015;115:8B–21B. 10.1016/j.amjcard.2015.01.035 25697919

[26] Ho JE, Chen W-, Chen M-, Larson MG, McCabe EL, Cheng S et al Common genetic variation at the IL1RL1 locus regulates IL-33/ST2 signaling. *J Clin Invest* 2013;123:4208–4218. 10.1172/JCI67119 23999434 PMC3784527

[27] Saunders JT, Nambi V, De Lemos JA, Chambless LE, Virani SS, Boerwinkle E et al Cardiac troponin T measured by a highly sensitive assay predicts coronary heart disease, heart failure, and mortality in the atherosclerosis risk in communities study. *Circulation* 2011;123:1367–1376. 10.1161/CIRCULATIONAHA.110.005264 21422391 PMC3072024

[28] Lyngbæk S, Winkel P, Gøtze JP, Kastrup J, Gluud C, Kolmos HJ et al Risk stratification in stable coronary artery disease is possible at cardiac troponin levels below conventional detection and is improved by use of N-terminal pro-B-type natriuretic peptide. *European Journal of Preventive Cardiology* 2014;21:1275–1284. 10.1177/2047487313492099 23723326

[29] Wong TC, Piehler K, Meier CG, Testa SM, Klock AM, Aneizi AA et al Association Between Extracellular Matrix Expansion Quantified by Cardiovascular Magnetic Resonance and Short-Term Mortality. *Circulation* 2012;126:1206–1216. 10.1161/CIRCULATIONAHA.111.089409 22851543 PMC3464491

[30] Junttila MJ, Barthel P, Myerburg RJ, Mäkikallio TH, Bauer A, Ulm K et al Sudden cardiac death after myocardial infarction in patients with type 2 diabetes. *Heart Rhythm* 2010;7:1396–1403. 10.1016/j.hrthm.2010.07.031 20682359

[31] Rockey DC, Bell PD, Hill JA. Fibrosis—A Common Pathway to Organ Injury and Failure. *N Engl J Med* 2015;372:1138–1149. 10.1056/NEJMra1300575 25785971

[32] Zinman B, Wanner C, Lachin JM, Fitchett D, Bluhmki E, Hantel S et al Empagliflozin, Cardiovascular Outcomes, and Mortality in Type 2 Diabetes. *N Engl J Med* 2015;373:2117–2128. 10.1056/NEJMoa1504720 26378978

